# Optical diffraction for measurements of nano-mechanical bending

**DOI:** 10.1038/srep26690

**Published:** 2016-06-03

**Authors:** Rodolfo I. Hermans, Benjamin Dueck, Joseph Wafula Ndieyira, Rachel A. McKendry, Gabriel Aeppli

**Affiliations:** 1 London Centre for Nanotechnology, University College London, 17-19 Gordon Street, London WC1H 0AH, UK; 2 Department of Physics and Astronomy, University College London, Gower Street, London WC1E 6BT, UK; 3 Leica Camera AG, Am Leitz-Park 5, 35578 Wetzlar, Germany; 4 Jomo Kenyatta University of Agriculture and Technology, Department of Chemistry, PO Box 62000, Nairobi, Kenya; 5 Division of Medicine, Rayne Institute, 5 University Street, London WC1E 6JF, UK; 6 Departments of Physics, ETH Zürich, CH-8093 Zürich, Switzerland and École Polytechnique Fédérale de Lausanne (EPFL), CH-1015 Lausanne, Switzerland; 7 Synchrotron and Nanotechnology Department, Paul Scherrer Institute, CH-5232, Villigen, Switzerland

## Abstract

We explore and exploit diffraction effects that have been previously neglected when modelling optical measurement techniques for the bending of micro-mechanical transducers such as cantilevers for atomic force microscopy. The illumination of a cantilever edge causes an asymmetric diffraction pattern at the photo-detector affecting the calibration of the measured signal in the popular optical beam deflection technique (OBDT). The conditions that avoid such detection artefacts conflict with the use of smaller cantilevers. Embracing diffraction patterns as data yields a potent detection technique that decouples tilt and curvature and simultaneously relaxes the requirements on the illumination alignment and detector position through a measurable which is invariant to translation and rotation. We show analytical results, numerical simulations and physiologically relevant experimental data demonstrating the utility of the diffraction patterns. We offer experimental design guidelines and quantify possible sources of systematic error in OBDT. We demonstrate a new nanometre resolution detection method that can replace OBDT, where diffraction effects from finite sized or patterned cantilevers are exploited. Such effects are readily generalized to cantilever arrays, and allow transmission detection of mechanical curvature, enabling instrumentation with simpler geometry. We highlight the comparative advantages over OBDT by detecting molecular activity of antibiotic Vancomycin.

Micro-cantilevers are the most widely deployed micro-mechanical system (MEMS), initially developed for atomic force microscopy^1^, but now serving as ultra-sensitive force transducers for applications ranging from airbag release to motion detection in mobile telephones. They have enabled nanobiotechnology^2^,^3^, branching beyond imaging into single-molecule manipulation and force metrology^2^,^4^, as well as multifunctional lab-on-a-tip^2^ techniques. Cantilevers are promising for future medical diagnostic devices because they are both sensitive, with unlabeled biomolecules detected down to femtomolar concentrations within minutes^5^,^6^, and because they can be multiplexed on arrays that allow multiple simultaneous differential measurements^7^,^8^. The biochemical sensitivity of cantilevers derives from the ability to detect small motions of their untethered ends, usually via the optical beam deflection technique (OBDT)^9^,^10^ implemented extensively for AFM-like devices. While conceptually simple, the need for careful alignment by specialists and a laser spot size small compared to the dimensions of the cantilevers limit general applicability outside of specialized research laboratories as well as the miniaturization needed both for enhanced sensitivity and massive multiplexing.

One reason for the preeminence of OBDT is that when the first atomic force microscopes (AFM) were developed 30 years ago, inexpensive digital imaging (DI) was unavailable. Current ubiquity of DI makes the adoption barrier negligible. In this paper, we describe how cheap DI enables a much more robust method, namely far field diffractive imaging, for optical readout of cantilever arrays. The method operates with light beams which can be much larger than individual cantilevers and whose angle of incidence and reflection need not be precisely set and measured, thus removing the obstacles presented by OBDT for non-expert use, miniaturization and multiplexing, and thus opening optically readout cantilever arrays to numerous applications outside specialist research laboratories. It relies on the interference fringes easily visible for all objects with features on the scale of the wavelength of light, and we illustrate it for ordinary cantilevers, where the fringes are derived from their edges, as well as cantilevers into which we have inserted-using focused ion beam prototyping – periodic arrays of slots to create gratings whose diffraction patterns are very sensitive to bending.

The scientific literature describes a variety of cantilever metrology techniques based on diffraction and interference. Interferometry techniques have been implemented using optical fibre^11^ or microscope objectives^12^ making the method highly sensitive to misalignments. Some rely on diffraction gratings^13^,^14^ or interdigital structures^15^,^16^,^17^. Existing work using DI have limited themselves to calculating centroid positions ignoring any observed diffraction patterns^18^. The new technique we describe differs significantly from previous methods in several aspects, including a more sophisticated exploitation of inexpensive DI and total dispensation with any reference beam. It depends fundamentally on an observable that is invariant to translation and rotation and on the ability to decouple the measurement of tilt and curvature of the cantilever.

The paper starts with a description of design constraints and interference effects associated with all-optical readouts of cantilever bending, and then describe our tests of the diffractive method for unpatterned and patterned cantilevers, first for remote temperature sensing and then for biomedicine, where we examine antibiotic action.

## Huygens-Fresnel description

**Figure 1:**
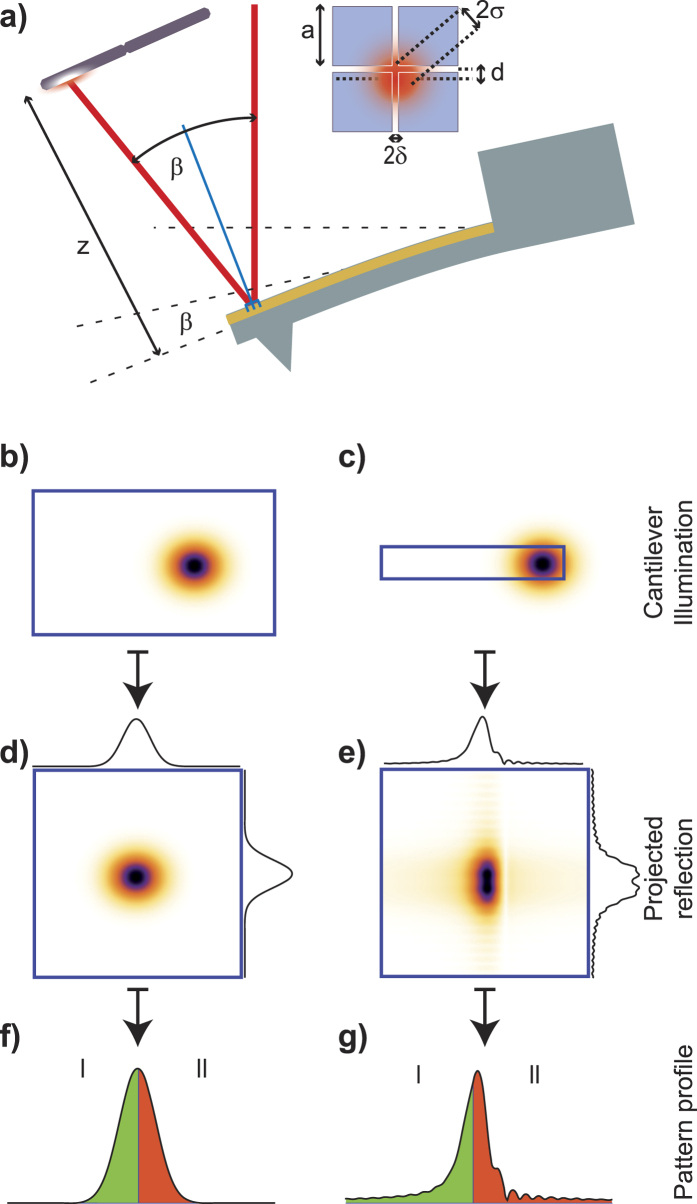
(**a**) Operation principle of the classical optical beam deflection technique (OBDT): Bending of the cantilever changes the reflection angle of a light beam. The reflected beam is projected onto a split photo-detector (also in inset) and the top-bottom differential signal is assumed proportional to the bending (See equation SE14). (**b**) Ideal case, a cantilever is illuminated by a beam much smaller than its width, causing a Gaussian reflected beam to be projected on to the split photo-detector (**d**). (**f**) The projected profile is symmetric as is therefore also the sensitivity curve. (**c**) Non-ideal case, the cantilever edges are illuminated causing (**e**) a cross-shaped diffraction patter projected onto the split photo-detector. (**g**) The projected profile as well as the sensitivity curve are asymmetric.

Optical techniques for MEMS metrology require the illumination of a region or all of the device probed. We focus our attention on the details of OBDT depicted in Fig. 1a, whose operation consists of reflecting a focused or collimated laser beam from the free end of a cantilever and measuring the position of the reflection projected onto a segmented photo-diode or similar position-sensitive device.

We model the optical system using the Huygens-Fresnel principle, where the light re-emitted by the cantilever, both reflected or transmitted, can be understood as the summation of an infinite number of infinitesimal point sources located on the cantilever surface. A cantilever acts as a rectangular slit source, a finite region emitting light in the plane 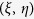 with a phase delay given by its curvature. We study Fresnel’s approximation for the optical wave in the observing plane (*x*,*y*) for a given geometry and illumination. The beam projected onto the device is assumed to have a 2D Gaussian profile of size *σ* centred at the origin.

### Ideal infinite plane

In the ideal case of a perfectly flat, infinite reflecting surface, the incident beam will be reflected unperturbed into a detector, maintaining the original profile. We model the finite size of the detector and consider the Gaussian beam projected on to a 4-segment photodiode of size *a* with gaps 2*δ* and calculate the differential signal *V* of the segmented sensor versus beam displacement *d* (See Fig. 1a for diagram and supplementary material for derivation of exact solution, approximations, analysis, and further figures).

We find that approximating the measured signal as proportional to the beam displacement (and therefore to the cantilever curvature) (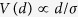) implies maximum gain and linearity; and it is valid within 1% only if 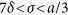, and 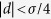. We observe in equation (SE14) that a smaller spot size *σ* appears to increase the gain, as previously reported^19^,^20^, but it must be noted that this is true only if *δ* is much smaller than *σ*. Because the signal is linear within 1% only for 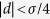 a small laser spot size will also restrict the measuring dynamic range.

For beams not collimated but focused at a finite distance, 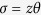 and 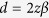 (See Fig. 1a where *z* is the detector distance, *β* is the cantilever deflection angle, and *θ* the beam divergence) and therefore the signal proportional to 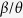 is independent of *z* and is maximized by reducing the beam divergence *θ*. Considering that the minimum beam cross-section diameter is given by 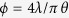
21, we see that a small beam divergence determines the minimum size of the laser spot and consequently the minimum width of the cantilever, if diffraction is to be avoided, as we will see.

### Implications for standard readout

The infinite plane model could only be valid if the reflected beam cross-section is completely contained in a flat reflecting surface (Fig. 1b), otherwise any illuminated edge of the cantilever (Fig. 1c) will cause a diffraction pattern to appear on the photo-detector.

**Figure 2:**
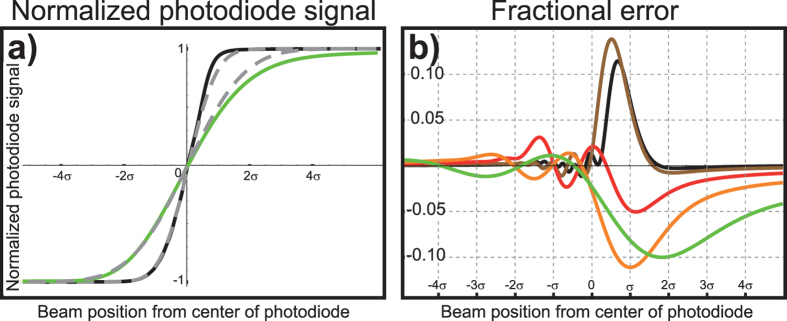
(**a**) Normalized differential photodiode signal versus beam position (assumed proportional to cantilever bending) for an infinite detector at different observation distances, in solid lines considering the diffraction caused by the cantilever edge and in dashed lines neglecting diffraction. (**b**) Magnitude of the fractional error as a function of beam position in units of *σ* for different conditions *λz*/*σ*^2^ = 1, 2, 5, 10 and 20 in colours black, brown, red, orange and green respectively. The error as a consequence of neglecting the diffraction phenomena can surpass 10% in some cases.

Figure 1d,e shows calculated diffraction patterns caused by a Gaussian beam reflected from cantilevers with edges respectively far and close to the beam centre. Illuminating the cantilever’s edge causes a broad-tailed asymmetric diffraction pattern, significantly different from the typically assumed Gaussian intensity distribution. This diffraction artefact causes an asymmetric dependence of the measured signal on the cantilever deflection. As shown in Fig. 2a, for negligible diffraction the curve is the Error function, symmetric around zero, but for considerable diffraction that symmetry is lost. The artefact is trivial for controlled systems, where a feed-back loop maintains the cantilever bending at a small constant value and the excursions from this value are small during experimentation. Interestingly, the asymmetry of the sensitivity could become relevant for uncontrolled systems, such as bio-markers, and systems where calibration and measurement happen at opposite sides of the deflection curve. For instance, in single-molecule force-spectroscopy experiments, signal versus cantilever bending calibration curves are obtained from upward-bending (pushing the cantilever into the surface) long trajectories whereas sample measurements are downward-bending for pulling^4^,^22^. Fig. 2b shows the normalized difference between considering or neglecting diffraction. The differences between the diffraction-calculated curve and the Error function are shown in Fig. 2b in normalized units of *σ* (the size of the illuminating beam at the detector) and for different magnitudes of the dimensionless parameter *λz*/*σ*^2^. Estimations of curvature changes or cantilever displacement could therefore be under or overestimated by more than 10%.

We have seen that the ideal case for OBDT is the limit where the illuminating beam is much smaller than the cantilever. In practice, small numerical aperture systems create focal spots comparable in size to cantilever widths (10–100 *μm*). Carefully aligning and focusing a small laser spot onto the centre of a cantilever end, to avoid the diffraction caused by the edges, can become a tedious task, if at all possible. We investigate the opposite limit, where the illuminated area is much bigger than the cantilever, and the diffraction pattern caused by the finite cantilever size contains all the information we need.

## Cantilever diffraction detection method

**Figure 3:**
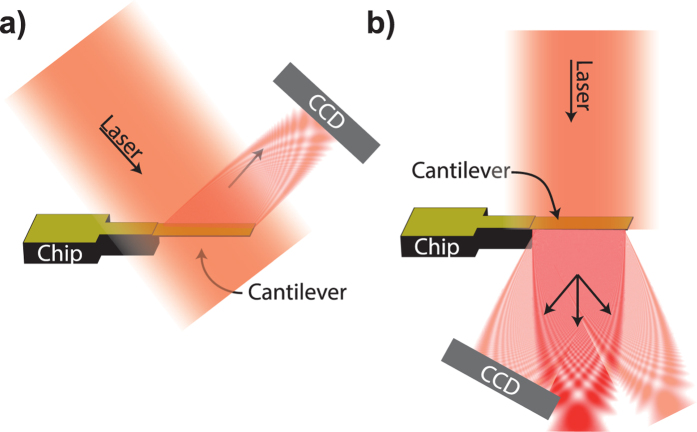
Two possible diffraction read-out operation modes. (**a**) The light reflected from the finite size cantilever causes a diffraction pattern. (**b**) The light transmitted through the cantilever causes the diffraction pattern. Even though typical transmittance of solid cantilevers could be insufficient to provide an acceptable signal to noise ratio in transmission mode, the fact that the features of interest in the diffraction pattern *i.e.* position shift and magnification are independent of cantilever shape, any arbitrary pattern of holes through the cantilever would allow higher transmittance. In particular an array of slits provides the advantage of high intensity high order diffraction peaks detectable at wide angles away from the direct incident beam.

Now we demonstrate that the shape of the diffraction pattern reveals the details of surface curvature. Figure 3 shows a cartoon of two operational modes of the new proposed readout method. A broad laser source illuminates the whole cantilever with close to homogeneous intensity while a CCD or CMOS detector captures some of the diffraction fringes created by either reflected (Fig. 3a) or transmitted light (Fig. 3b)^23^.

In the supplementary material we demonstrate that in reflection mode (Fig. 3a) the diffraction pattern from a rectangular cantilever of dimensions (*w*, *l*) curved along the 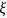 axis with shape given by a quadratic polynomial 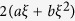 is related to the pattern of a straight cantilever by the transformation


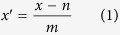


and observe that the term *n* = 2*az* causes a shift and *m* = 1 + 4*bz* a magnification of the diffraction pattern^24^. These results hold after applying the condition 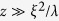, the Fraunhofer approximation for the far field. We see that also in the Fraunhofer far-field approximation the diffraction pattern caused by a micro-structure experiences a magnification given by the curvature of the surface and a shift in position given by the tilt of the surface as defined by the change of variables in equation (1). Therefore, tracking the changes in position and shape of a diffraction pattern enables to monitor the tilt and curvature of the cantilever independently. This result also holds for different cantilever shapes in reflection mode.

To model the transmission mode (Fig. 3b), we took a step back and considered the Fresnel approximation not in a plane but from a curved source. We follow the same procedure as before but with *z* replaced by 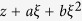. We approximate up to second order in the numerator exponent and re-arrange and find a similar transformation


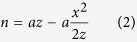



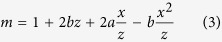


To the extent that 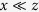 we can neglect the terms containing *x* in the right hand side and recover a similar result as before, this time only for small changes in cantilever tilt and curvature, implying a pattern shift of *az* and pattern magnification of 1 + 2*bx*, respectively. The magnitudes differ from the previous result by a factor of two because, for a given cantilever displacement, light travels the path only once in transmission mode but twice in reflection mode.

The ideal resolution for tilt *a* and curvature *b* estimations is expected to be of the order of 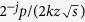 and 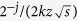 respectively, where *p* is the pixel size, *j* is the effective number of bits of digitization resolution, *k* is the diffraction pattern size in units of linear number of pixels in the detector and *s* is the number of samples.

We next explore experimentally both the case of a flat cantilever and also the case where the cantilever has a series of narrow slits forming a diffraction grating.

**Figure 4:**
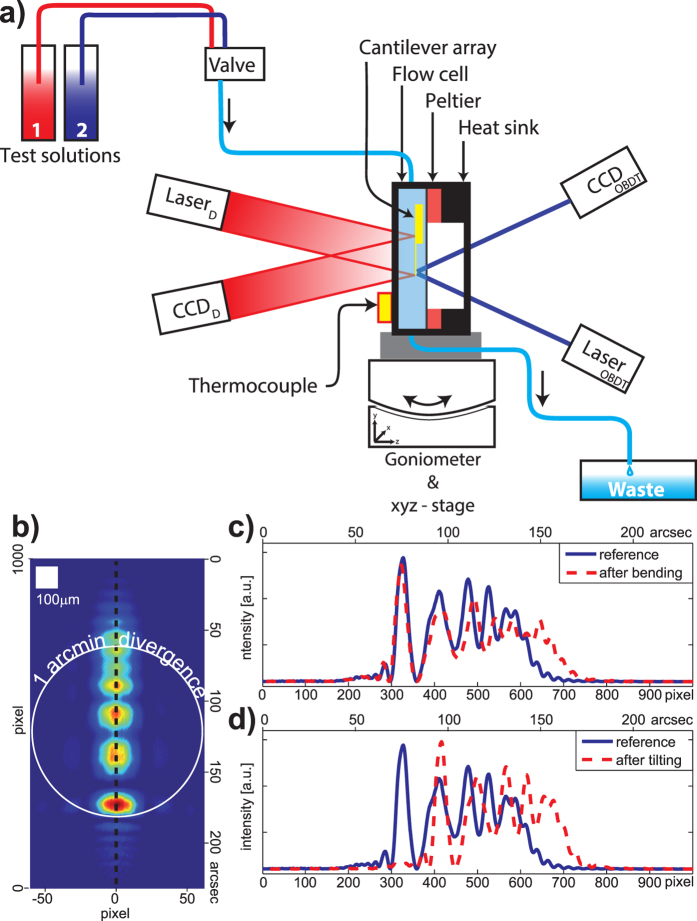
(**a**) Schematic design of the experiment. The cantilever is mounted vertically in the flow cell. Test solutions 1 and 2 are selected by a valve and driven through the cell into the waste container by gravity flow. On the right side, the optical beam deflection technique (OBDT) is used to read out the cantilever bending and on the left side is the new diffractive readout in reflection mode consisting of broad beam HeNe Laser illumination (632.8 nm, 5.0 mW HRR050 Thorlabs) and CCD detection. Goniometer (RV160CCHL, VP-25X from Newport) and CCD are controlled by one LabView program. The TCM controller for the Peltier module and the pico-logger for the external thermocouple were controlled by two separate programs. (**b**) Reflection-mode 2D diffraction pattern from a flat cantilever captured by a CCD ORCA-AG Hamamatsu (**c**) Comparison of the cross-section of reference and measured pattern after a change in curvature (Temperature change from 25 °C to 24 °C) and (**d**) after tilting approximately 25 arcsec.

To verify the usefulness and performance of this detection method we have built the trial setup of Fig. 4a. We used a cantilever array in a windowed flow cell that allowed simultaneous measurements using the new diffractive readout method and the classic OBDT. We capture diffraction patterns generated by the cantilevers and study the changes as the cantilever rotates (goniometer tilt) and curves (temperature change). Fig. 4b shows the 2D pattern acquired with a CCD. Figure 4c,d shows the pattern profile and confirms experimentally our prediction that, independent of the details of the pattern, changes in curvature magnify the pattern profile and changes in tilt only displace the pattern without significant deformation.

**Figure 5:**
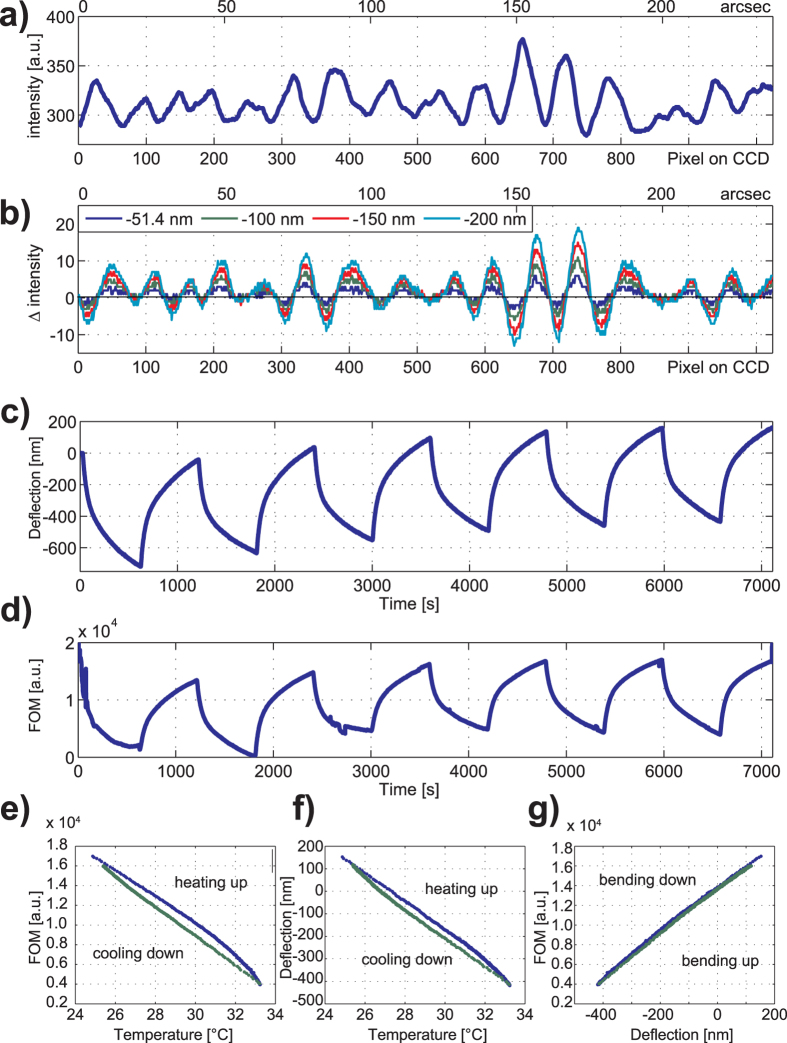
Deflection measurements with non-patterned cantilevers. (**a**) Cross-section intensity of a diffraction pattern is used as a reference. (**b**) Four representative differences between the observed and reference pattern are calculated for cantilever deflections during a temperature cycle (inset distances via OBDT correspond to changes in temperature of approximately 0.7 °C, 1.4 °C, 2.0 °C and 2.8 °C respectively). (**c**) Cantilever deflection measured by OBDT as a function of time while the cantilever is cycled in temperature from 25 °C to 33 °C. (**d**–**g**) The figure of merit (FOM) calculated as the root mean squared value of the pattern differential features an excellent correlation with the deflection measured by OBDT.

To exemplify the data acquisition procedure Fig. 5 shows measurements for the cantilever as the temperature is cycled in the range 25 °C to 33 °C. The gold-coated silicon cantilever acts as a bimetallic strip and the differential thermal expansion causes a homogeneous curvature of significant magnitude^25^. The observed deflection is around 72 nm/°C. A reference diffraction pattern is recorded by the CCD camera at the beginning of the experiment (Fig. 5a) as a spatial array of intensities. At the same time the initial position of the OBDT spot in the CCD is recorded also as a reference. All further patterns and spot positions are measured sequentially in time and compared with their respective references. The difference between the pattern intensity arrays are calculated (Fig. 5b). We define a figure of merit (FOM) as the root mean square value of the difference between the observed pattern and the reference pattern (Fig. 5b).


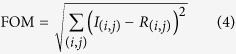


Figure 5g shows that the FOM calculated from the diffraction pattern closely correlates with the measurements from OBDT evidencing that the far-field diffraction readout can replace the classic OBDT. We observed a resolution of 0.95 nm/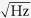 at normal video rate, or 0.47 nm at 100 frames per second.

**Figure 6:**
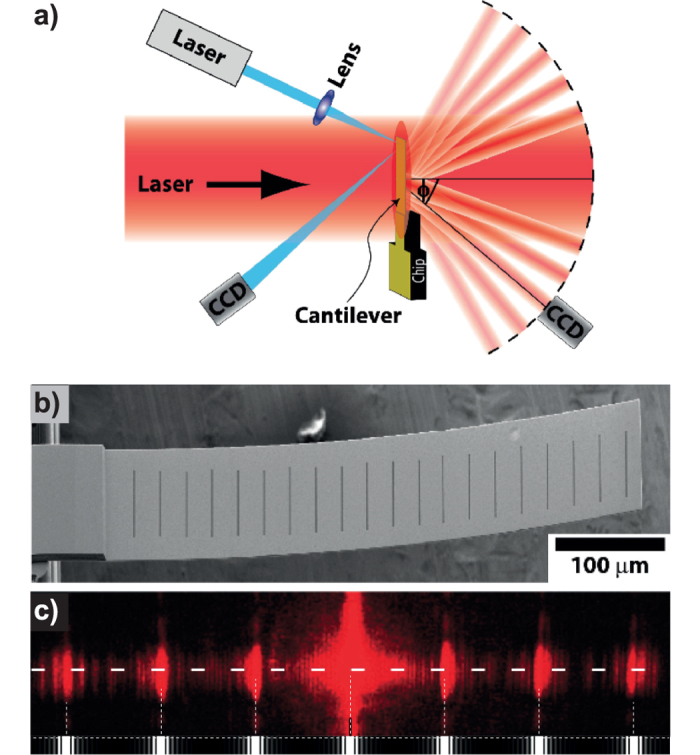
(**a**) Transmission mode experimental configuration: a broad laser beam illuminates the whole cantilever for diffraction read-out while simultaneously a narrow focused laser illuminates the cantilever tip for the optical beam deflection technique (OBDT). (**b**) A cantilever featuring an array of slits created by Focused Ion Beam (width *w* = 1 *μm*, spacing *s* = 23.4 *μm*) can act as diffraction grating allowing detection using high order Bragg peaks. (**c**) Measurement of a series of Bragg (strong red) and Fraunhofer (weak red) fringes created by the illuminated slit array compared with predicted fringes (white) by the diffraction model for a straight cantilever 
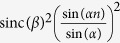
 with 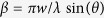, 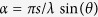, *n* = 20 and *λ* = 632.8 *nm*.

**Figure 7:**
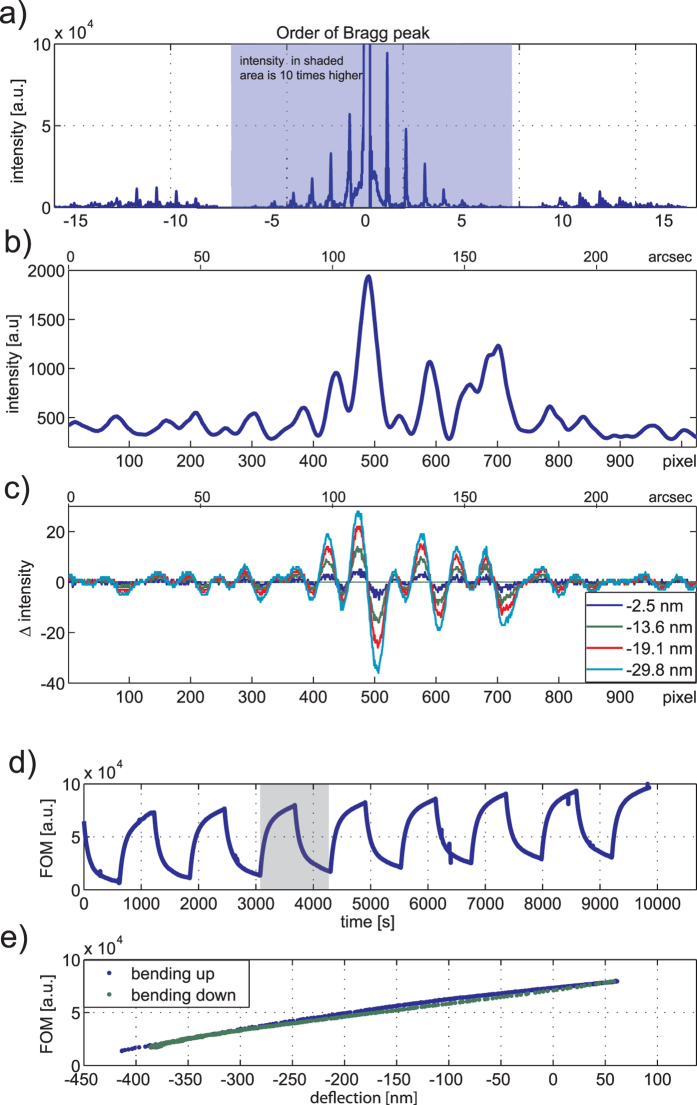
Transmission measurements with patterned cantilevers. (**a**) Diffraction pattern from −15th to +15th order. The intensity plotted in the shaded area has been displayed reduced by a factor of 10 to increase the visibility of higher order Bragg peaks. (**b**) Initial diffraction pattern showing the 19th order Bragg peak and subsidiary peaks. (**c**) The difference of diffraction patterns to the initial pattern where the bending is relative to the initial bending. (**d**) The response of the cantilever to cycling the temperature by approximately 5 °C. Figure of merit (FOM) computed from diffraction pattern. (**e**) FOM versus deflection as measured with OBDT.

Previous results can be reproduced both in reflection and transmission mode, but the latter may suffer from a poor signal to noise ratio if the cantilever features a small transmissivity. An interesting consequence of our analysis is that, where the approximations hold, the principle of diffraction readout applies independent of the form of the cantilever. In particular it applies also to a periodic array of slits. We performed a second experiment with a cantilever featuring a series of narrow slits created by the Focused Ion Beam technique as shown in Fig. 6b. Here the light transmitted through the array of slits causes an intense series of Bragg peaks (Figs 6c and 7a) with spacing reciprocal in relation to the spacing of the slits. The weaker Fraunhofer peaks between the Bragg peaks result from the finite number of slits. More slits will increase the number of Fraunhofer fringes and decrease their intensity. The entire feature-rich pattern is sensitive to the phase differences caused by cantilever deflection and consequently, contrary to other techniques, a patterned cantilever allows the detection of bending across a broad range of detection angles.

**Figure 8:**
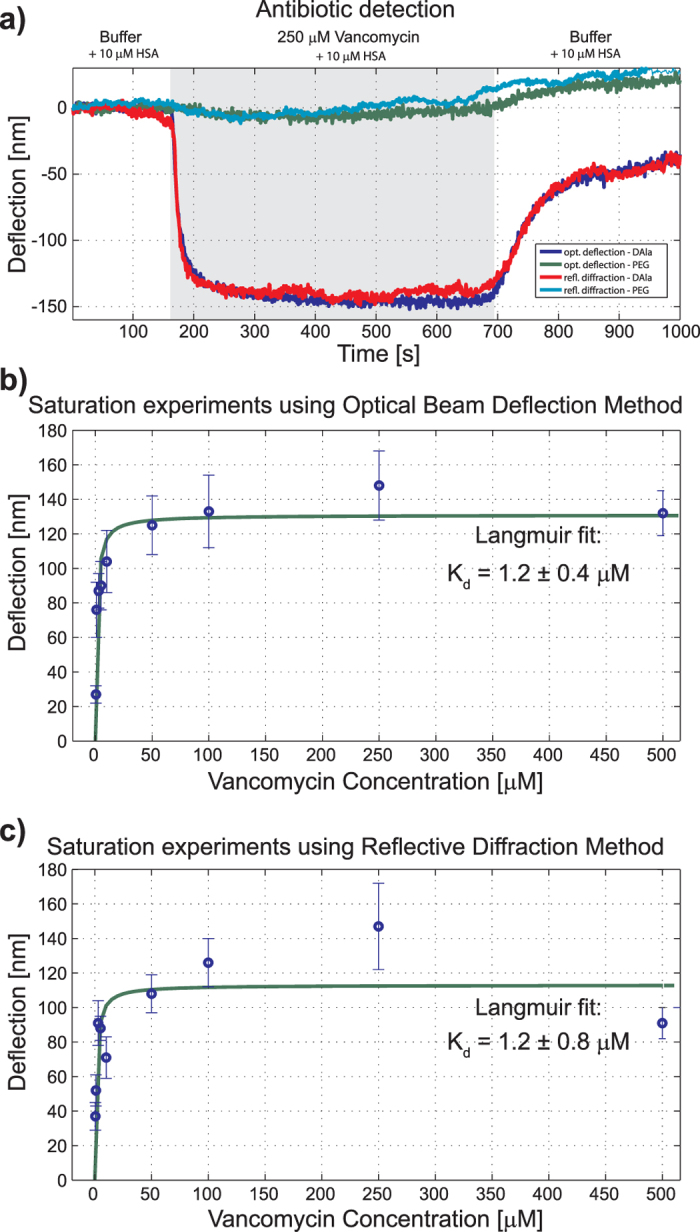
Experimental estimation of dissociation constant of Vancomycin via saturation experiments in cantilevers is performed simultaneously with both techniques, reflective diffraction and OBDT. (**a**) The dramatic bending observed on the functionalized cantilever is attributed to the surface pressure caused by the specific ligand-receptor binding of Vancomycin and a bio-mimetic bacterial cell wall target. In contrast, passivated cantilevers feature negligible bending. Equal equilibrium bending is estimated both by OBDT and diffraction detection techniques. The equilibrium deflection measured at different concentrations of Vancomycin are fitted by a Langmuir isotherm function. Identical values of the dissociation constant *K*_*d*_ are obtained using (**b**) OBDT and (**c**) our diffraction technique. Data points are mean values and error bars standard deviation, with *n* = 3.

To further test the method in challenging conditions of practical interest we reproduce previous results on binding of antibiotics to target peptides^8^. Un-patterned cantilevers were either sensitized or passivated by selectively forming a self-assembled molecular monolayer by individual incubation in micro-capillary tubes. Passive control cantilevers were coated with polyethylene glycol (PEG) and target cantilevers were coated with drug-sensitive mucopeptide analogue in a procedure detailed elsewhere^8^. Figure 8a shows bending of cantilevers coated with a bio-mimetic bacterial cell wall target in response to 250 *μ*M Vancomycin detected with both the diffractive method and OBDT. Upon repetition at different Vancomycin concentrations we obtain the saturation curves in Fig. 8b,c featuring identical dissociation constants, irrespective of the detection method. The variations on the captured traces are consistent with previous experiments presented in the literature^8^ and interpreted to be a consequence of small instabilities of the physical system (incuding particularly microfluidics) and significantly bigger than the intrinsic resolution of the measurement methods.

## Advantages and perspective

Diffraction features generated by a microstructure such as a cantilever are exquisitely sensitive to geometrical details such as curvature, tilt, position of the edges and roughness of the surface^24^. We have shown that this sensitivity can on one hand yield artefacts that slightly skew assumed calibrations of OBDT. On the other hand, they provide alternative means for detecting independently changes of tilt or curvature. Avoiding diffraction from the cantilever surface requires comparatively narrow illuminating beams or broad flat reflection areas. If it exists at all, the optimum position and focus of the illuminating beam will tend to be narrow, and therefore continuous and tedious re-alignment and re-calibration could be necessary. Another potential problem of highly focused laser beams in liquid (often ignored) is that the measured intensity becomes sensitive to transient perturbations caused by aggregates or other impurities drifting across the beam at the narrow focus, be they suspended in the liquid or diffusing in the cantilever surface. There are several advantages to be gained by measuring the details of diffraction patterns, instead of trying to avoid them.

Keeping the laser spot larger than the cantilever dimensions makes crucial calibration adjustments, such as the exact knowledge of the laser spot position^26^, superfluous and therefore alignment becomes a fast, simple and reliable procedure. A broad illumination beam also minimizes the relative magnitude of perturbations caused by particles crossing the illumination beam and eliminates temperature gradients in the cantilever^25^,^27^,^28^. The optical diffractive readout does not necessarily rely on having a reflective surface and therefore the choice of surface coatings is widened. Beside the reflection component, cantilevers featuring slit arrays allow high signal-to-noise levels in transmission mode, and more interestingly, the detector can be located off-axis around high order Bragg peaks, offering a much broader set of geometrical configurations for the detector. The relative change in size of the diffraction pattern is of particular interest because it is invariant to lateral translation and rotation and independent of the shift caused by changes in tilt, making the measurement intrinsically robust to small perturbations in the detector and cantilever positions and orientations.

We have provided an exact analytical model for parabolic bending and reflection mode and an approximate analytical solution for transmission mode when 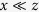. We have also defined a figure of merit (FOM) that allows an effective implementation of the detection technique independent of these analytical considerations or other modelling.

The high order Bragg fine structure resembles that exploited for oversampled X-ray crystallography^29^. We have a visible light analogue of the X-ray experiments and here the information from the phase difference created by the cantilever curvature is contained in the details of the intensity between Bragg peaks.

Measuring the details of a diffraction pattern requires a more complex detection device such as a CCD or CMOS sensor array, as opposed to a simpler split photodiode. This increase in complexity is justified by the increased amount of information available, as tilt and bending is simply decoupled, in significant contrast to OBDT where there is no shape information. CCD and CMOS sensor also feature reduced bandwidth, but this is not a limitation for probing systems with relevant time scales much longer than the CCD frame acquisition period, such as the ones shown here. It is also worth noting that the ubiquity of digital imaging today, especially as compared to when OBDT was developed in the early 1990s, makes the use of position-sensitive optical detection a very competitive option for modern low-cost instrumentation.

The presented far-field technique distinguishes itself from the related NANOBE^24^ in many forms, including that it does not demand a lens to maintain the near field condition, allows both reflection and transmission mode for patterned and un-patterned cantilevers, it exploits high order Bragg fine structure similarly to over-sampled X-ray crystallography, permits off-axis detection, by working in the far-field and not near-field. In addition, it can operate in modes either sensitive to single cantilever deflection or to differential displacement, depending on the illumination profiles. In the current work we have also demonstrated an independent model-free FOM measurable that perfectly correlates with OBDT. At the far field, if more than one cantilever is illuminated at a time, the observed diffraction pattern will be sensitive to the differential displacement, while in the near field there is negligible overlap between information from near cantilevers.

In summary, we have analysed mathematically the popular optical beam deflection technique (OBDT) for measuring cantilever deflection and found that the conditions for maximum gain, linearity and symmetry require illumination spot sizes that are heavily constrained by the geometry of the detector and the cantilever, desired dynamic range and gain, and the divergence of the illuminating beam. Ignoring such constraints can cause detection errors in excess of 10%, acknowledging such constraints provides robust instrumentation design guidelines not previously available in the literature. These considerations are likely to be of concern mainly for cantilevers that undergo significant changes in curvature, such as for biosensors and in single-molecule force spectroscopy.

We propose a diffraction readout method which decouples as independent observables the cantilever tilt and curvature of the cantilevers and does not require precise alignment. The excellent correlation observed (Fig. 5g) between the OBDT and our proposed diffraction method demonstrates similar performance at ideal conditions. The advantages of replacing OBDT with the more robust diffraction method comes not from an increased performance at ideal conditions but from the special features of the technique *i.e.* from the intrinsic resilience to various artefacts, mainly the misalignments of the optical components and from the ability to inform about the shape details, decoupling tilt from curvature. While specialized instrumentation operated by trained scientists in ideal conditions, such as imaging AFM, may not benefit directly from this technique, the combination of unique attributes of the diffraction technique makes it especially suitable to bring cantilever technology to the consumer market for bio-sensing applications and related fields. We have demonstrated the fundamental principles and practicality of our approach for a clinically relevant application. The approach taken could enable not only more robust pharmacological research instruments as decribed here, but even portable medical diagnostic tools, featuring high performance without specialist operators.

## Methods

### Readout

We used a cantilever array chip (IBM) where each cantilever was 500 *μ*m long, 100 *μ*m wide and 0.9 *μ*m thick, and coated with a layer of 2 nm titanium followed by 20 nm of gold. The array chip was mounted in an aluminium flow cell with sapphire windows at both sides and a thermoelectric Peltier element and thermocouple for temperature control. A broad laser beam (HeNe 632.8 nm, 5.0 mW, HRR050 Thorlabs)) illuminates the surface of a single cantilever to test the diffractive readout method. Simultaneously, a narrowly focused laser beam was used to measure the cantilever bending using OBDT as control. Both reflected beams were projected onto CCD sensors (ORCA-AG from Hamamatsu, Pixel size: 6.45 *μ*m × 6.45 *μ*m and FireWire 400 Color Industrial Camera DFK 31AF03 with sensor Sony ICX204AK, Pixel size 4.65 *μ*m × 4.65 *μ*m) mounted on calibrated goniometers (Rotation Stage RV160CCHL and xyz-stage VP-25X from Newport) to allow recording the intensity at different angles. CCD sensors were approximately at a distance of 100 mm for reflection mode and 250 mm for transmission mode. Our raw data consist of the diffraction patterns generated by the cantilevers captured as 12 bit TIFF images. Exposure times on the order of milliseconds were adjusted to avoid saturation and maximize dynamic range. The expansion of the pattern by approximately 12.5% observed in Fig. 4c corresponds to a change in curvature of 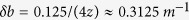 and a displacement at the end of the cantilever of 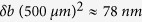.

## Additional Information

**How to cite this article**: Hermans, R. I. *et al*. Optical diffraction for measurements of nano-mechanical bending. *Sci. Rep.* **6**, 26690; doi: 10.1038/srep26690 (2016).

## Supplementary Material

Supplementary Information
