# Supplementary material for: Optical diffraction for measurements of nano-mechanical bending
Source: Sci Rep. 2016 Jun 3;6:26690. doi: 10.1038/srep26690 (PMC4891711; doi:10.1038/srep26690)
Supplement: Supplementary Information [file srep26690-s1.pdf]

Supplementary Information:
Optical diffraction for measurements of nano-mechanical bending

Rodolfo I. Hermans[1,2,*,+], Benjamin Dueck[1,3,+], Joseph Wafula Ndieyira[1,4], Rachel A. McKendry[1], and Gabriel Aeppli[1,2,5,6]

[1]London Centre for Nanotechnology, University College London, 17-19 Gordon Street, London WC1H 0AH, UK
[2]Department of Physics and Astronomy, University College London, Gower Street, London WC1E 6BT, UK
[3]Leica Camera AG, Am Leitz-Park 5, 35578 Wetzlar, Germany
[4]Jomo Kenyatta University of Agriculture and Technology, Department of Chemistry, PO Box 62000, Nairobi, Kenya
[5]Departments of Physics, ETH Zürich, CH-8093 Zürich, Switzerland and École Polytechnique Fédérale de Lausanne (EPFL), CH-1015 Lausanne, Switzerland
[6]Synchrotron and Nanotechnology Department, Paul Scherrer Institute, CH-5232, Villigen, Switzerland
[*]r.hermans@ucl.ac.uk
[+]These authors contributed equally to this work

ABSTRACT

Contents

SS1 Model for Photodiode Signal **SP2**
SS1.1 Infinite detector, no gap SP3
SS1.2 Finite-Size Detector SP3
SS1.3 Insensitive Gap SP4
SS1.4 Segmented photodiode conclusions SP5
SS2 Diffraction model **SP7**
SS2.1 Cantilever edge SP7
SS2.2 Curved cantilever SP7
Reflection • Transmission
References **SP9**

SS1 Model for Photodiode Signal

Four segment photo-diodes are ubiquitously used to implement the optical beam deflection technique. We can summarize the requirements for the geometry configuration of the photodiode and laser beam as $7\delta < \sigma < a/3$, and the operation range $d < \sigma/4$, where d is the distance of the center of the beam from the center of the detector, σ the standard deviation of the Gaussian beam at the detector, a is the length of the photosensitive square segments and 2δ is the distance between segments.. The following sections detail the method used to obtain these simple rules.

Let us assume that the laser beam distributes its total intensity I_{tot} in a 2D Gaussian with standard deviation σ. The intensity per unit area will be

$$I(x,y) = I_{\text{tot}} \frac{1}{2\pi\sigma^2} e^{-\frac{x^2+y^2}{2\sigma^2}} . \tag{SE1}$$

Assuming a photodiode detector with four square segment of size a. If the beam is centered in the y axe but off-centered in the x axe by a distance equal to d and if the detector have an insensitive region of width 2δ then the total intensity over one segment of the detector is I_A.

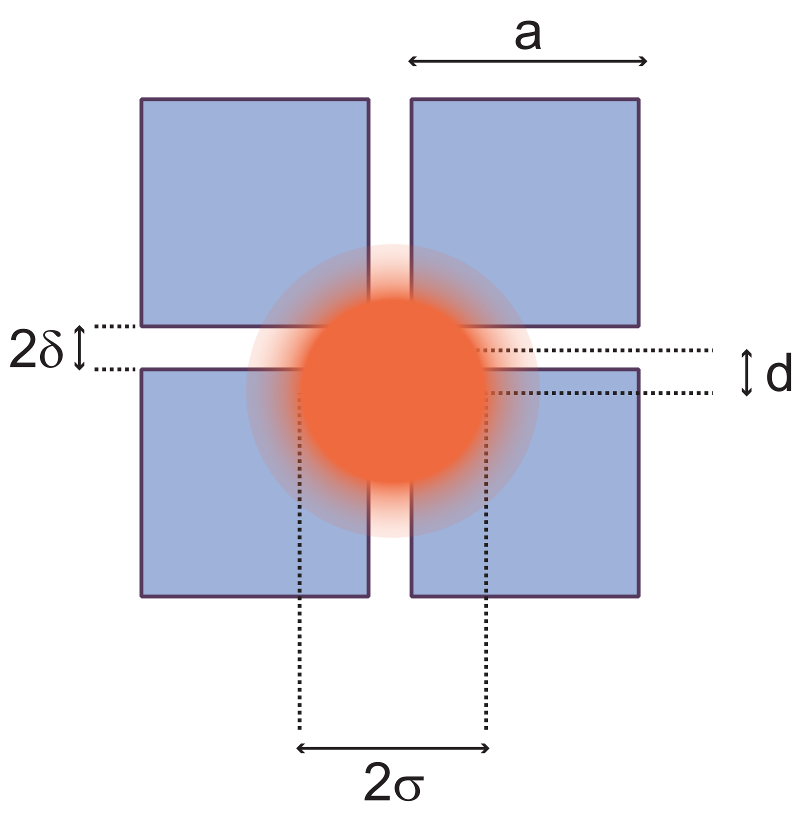

Figure SF1. Four quadrant photodiode can be modeled as four sensitive squares of edge length a separated by a insensitive gap 2γ. The projected beam is assumed to be have a 2D Gaussian distribution with standard deviation σ and centered at a distance d from the center of the detector.

$$I_A = I_{\text{tot}} \int_\delta^a \int_\delta^a \frac{1}{2\pi\sigma^2} e^{-\frac{(x-d)^2+y^2}{2\sigma^2}} dx\,dy . \tag{SE2}$$

To measure the displacement of the cantilever the quadrant photodiode provides a voltage proportional to $V_F \propto [(I_A + I_B) - (I_C + I_D)]$ that we write as a function of the geometry of the detector and the position of the reflected beam.

$$
\begin{aligned}
V_F &= G I_{\text{tot}} \frac{1}{2\pi\sigma^2} \left(\int_{-a}^{a} \int_{-a}^{-\delta} e^{-\frac{(x-d)^2+y^2}{2\sigma^2}} dx\,dy - \int_{-a}^{a} \int_{\delta}^{a} e^{-\frac{(x-d)^2+y^2}{2\sigma^2}} dx\,dy \right) \\
&= -G I_{\text{tot}} \frac{1}{2} \text{erf}\left(\frac{a}{\sqrt{2}\sigma}\right) \left[\text{erf}\left(\frac{a-d}{\sqrt{2}\sigma}\right) - \text{erf}\left(\frac{a+d}{\sqrt{2}\sigma}\right) + \text{erf}\left(\frac{d-\delta}{\sqrt{2}\sigma}\right) + \text{erf}\left(\frac{d+\delta}{\sqrt{2}\sigma}\right) \right].
\end{aligned}
\tag{SE3}
$$

Given the complexity of this expression an approximated form is desirable and an estimation of the range where the signal can be considered a linear function of the position. Here follows an analysis of the effect of the main geometrical features of the detector and the rage of parameters where the detector signal can be considered linear.

SS1.1 Infinite detector, no gap

We first analyze the case of limit where $\delta \to 0$ and $a \to \infty$ then equation SE3 can be written as

$$
\begin{aligned}
V_F(d) &= G I_{\text{tot}} \frac{1}{2\pi\sigma^2} \left(\int_{-\infty}^{\infty} \int_{-\infty}^{0} e^{-\frac{(x-d)^2+y^2}{2\sigma^2}} dx\,dy - \int_{-\infty}^{\infty} \int_{0}^{\infty} e^{-\frac{(x-d)^2+y^2}{2\sigma^2}} dx\,dy \right) \\
&= G I_{\text{tot}} \,\text{erf}\left(\frac{d}{\sqrt{2}\sigma}\right) \\
&\approx G I_{\text{tot}} \left[\sqrt{\frac{2}{\pi}} \left(\frac{d}{\sigma}\right) - \frac{1}{3\sqrt{2\pi}} \left(\frac{d}{\sigma}\right)^3 + \ldots \right].
\end{aligned}
\tag{SE4}
$$

Figure SF2 shows with a segmented line the exact solution and with solid line the linear approximation

$$
V_F \approx G I_{\text{tot}} \sqrt{\frac{2}{\pi}} \frac{d}{\sigma}. \tag{SE5}
$$

This approximation is exact within 1% provided that[1]

$$
d < \sigma/4 \tag{SE6}
$$

Equation SE6 provides the first requirement to obtain a photodiode signal $V_F(d)$ that is a linear function of the displacement d of the laser spot.

SS1.2 Finite-Size Detector

From equation SE3 for $\delta \to 0$, we observe that the effect of the finite size of the detector is to allow a portion of the light to fall outside of the sensitive region when σ is not negligible compared with a or $a \pm d$.

$$
V_F(d) = \frac{G I_{\text{tot}}}{2} \text{erf}\left(\frac{a}{\sqrt{2}\sigma}\right) \left[\text{erf}\left(\frac{a-d}{\sqrt{2}\sigma}\right) + 2\,\text{erf}\left(\frac{d}{\sqrt{2}\sigma}\right) - \text{erf}\left(\frac{a+d}{\sqrt{2}\sigma}\right) \right] \tag{SE7}
$$

$$
\approx G I_{\text{tot}} \sqrt{\frac{2}{\pi}} \left[\left(1 - e^{-\frac{a^2}{2\sigma^2}}\right) \text{erf}\left(\frac{a^2}{2\sigma^2}\right) \right] \frac{d}{\sigma} + \ldots \tag{SE8}
$$

The consequences of comparable sizes of detector and beam are: $V_F(d)$ is no longer a monotonic function and the magnitude of the gradient is diminished by the factor

$$
C = (1 - \exp(-a^2/2\sigma^2))\,\text{erf}(a^2/2\sigma^2). \tag{SE9}
$$

Figure SF3 shows with a segmented lines exact solutions for three different values of σ and with solid lines the corresponding linear approximations.

[1] Solved numerically $d = 0.244829\sigma$ and rounded to nicest rational.

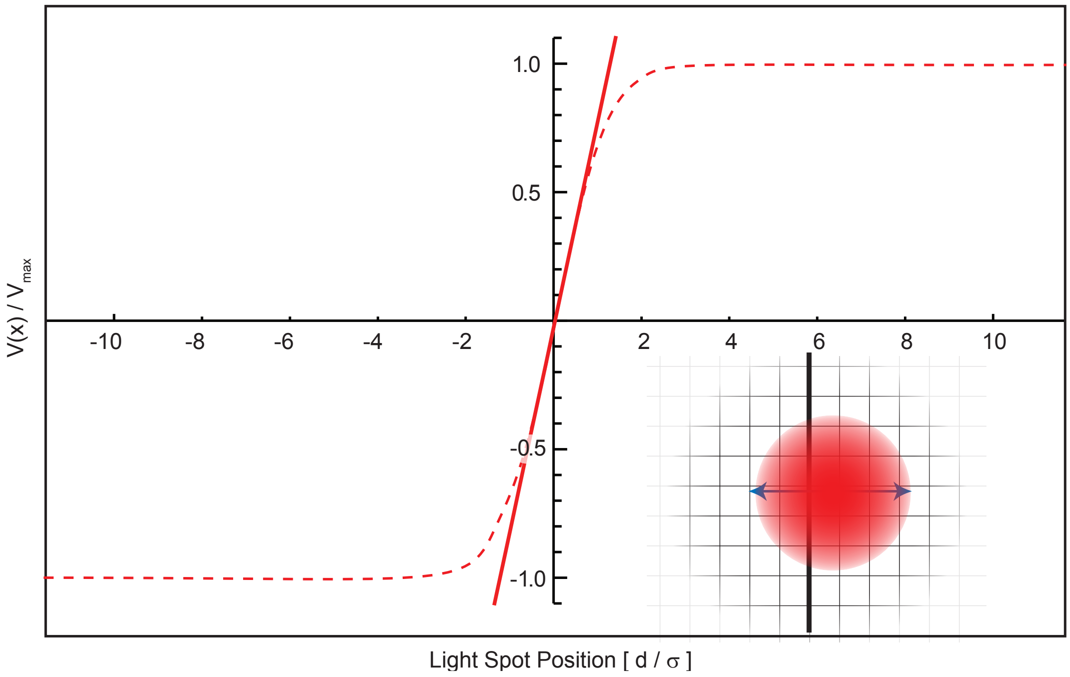

Figure SF2. Signal versus displacement for a detector of infinite size. Exact solution in segmented line, and linear approximation in solid line.

In general C can be approximated by

$$C \approx \left(1 + \frac{5}{2}\left(\frac{\sigma}{a}\right)^3\right)^{-1} \tag{SE10}$$

The factor C can be approximated[2] to $C = 1$ within 1% only when

$$a > 3\sigma\,. \tag{SE11}$$

Equation SE11 provides the second requirement to obtain a photodiode signal $V_F(d)$ that is a linear function of the displacement d of the laser spot.

SS1.3 Insensitive Gap

Again from equation SE3 we consider for simplicity $a \to \infty$ but now for a non-vanishing insensitive gap δ.

$$\begin{aligned} V_F(d) &= \frac{GI_{\text{tot}}}{2}\left(\operatorname{erf}\left(\frac{d+\delta}{\sqrt{2}\sigma}\right) + \operatorname{erf}\left(\frac{d-\delta}{\sqrt{2}\sigma}\right)\right) \\ &\approx e^{-\frac{\delta^2}{2\sigma^2}}\left[\sqrt{\frac{2}{\pi}}\frac{d}{\sigma} + \frac{(\delta^2-\sigma^2)}{3\sqrt{2\pi}\sigma^2}\frac{d^3}{\sigma^3} + \dots\right] \end{aligned} \tag{SE12}$$

we observe that the effect of the insensitive gap between two segments in the function $V_F(d)$ is to reduce the gradient at $d = 0$ by a factor of $\exp(-\delta^2/2\sigma^2)$ and when $\delta > \sigma$ to introduce two new inflexion points at $d \approx \pm\delta$. The effect of the gap is negligible within 1% when

$$\delta < \sigma/7\,. \tag{SE13}$$

[2]Solved numerically, $C = 0.99$ for $a = 3.10341\sigma$

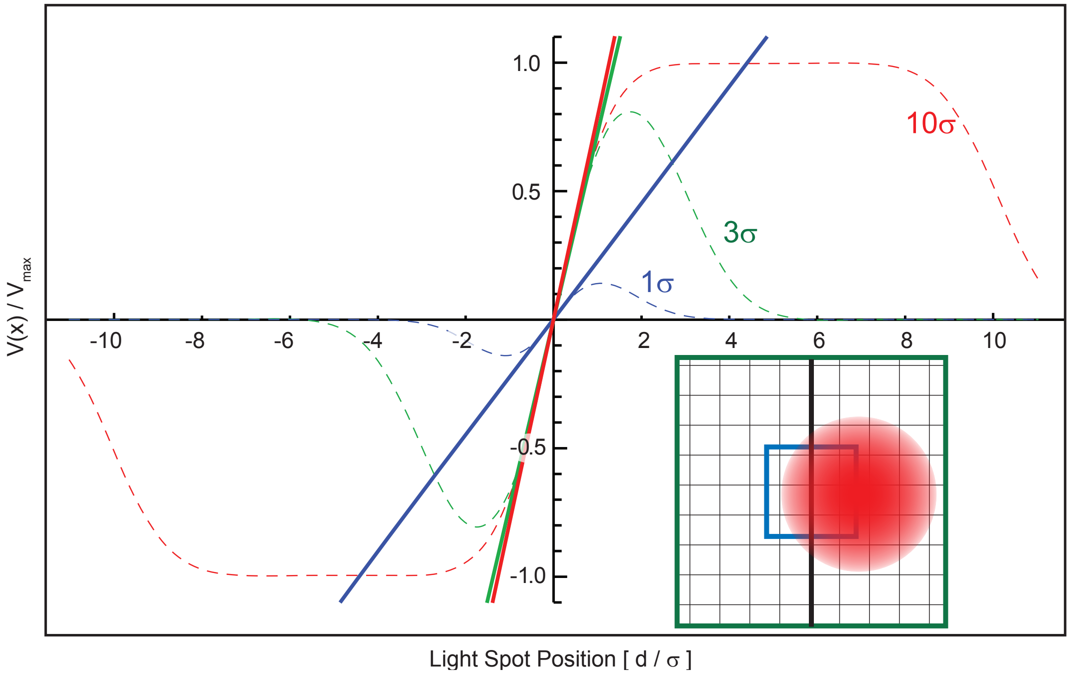

Figure SF3. Signal versus displacement for a detector of segment size $a = n\sigma$ for $n = 1, 3, 10$ in the colors red, green and blue. Exact solution in segmented line, and linear approximation in solid line. The effect of the detector size becomes considerable when $a < 3\sigma$

Equation SE13 provides the third and last requirement to obtain a photodiode signal $V_F(d)$ that is a linear function of the displacement d of the laser spot. Figure SF4 shows with a segmented lines the exact solutions for three different values of δ and with solid lines the corresponding linear approximations.

SS1.4 Segmented photodiode conclusions

We have offered details on the sensitivity curve relating the measured signal and the beam displacement in a segmented photodiode.

$$V(d) \approx G\frac{d}{\sigma}\,\mathrm{Erf}\left(\frac{a^2}{2\sigma^2}\right)\left[e^{-\frac{\delta^2}{2\sigma^2}}\left(1-e^{-\frac{a^2}{2\sigma^2}}\right)\right] \tag{SE14}$$

We concluded that linearity and maximum gain is obtained (within 1%) oly under the the constrains $7\delta < \sigma < a/3$, and $d < \sigma/4$.

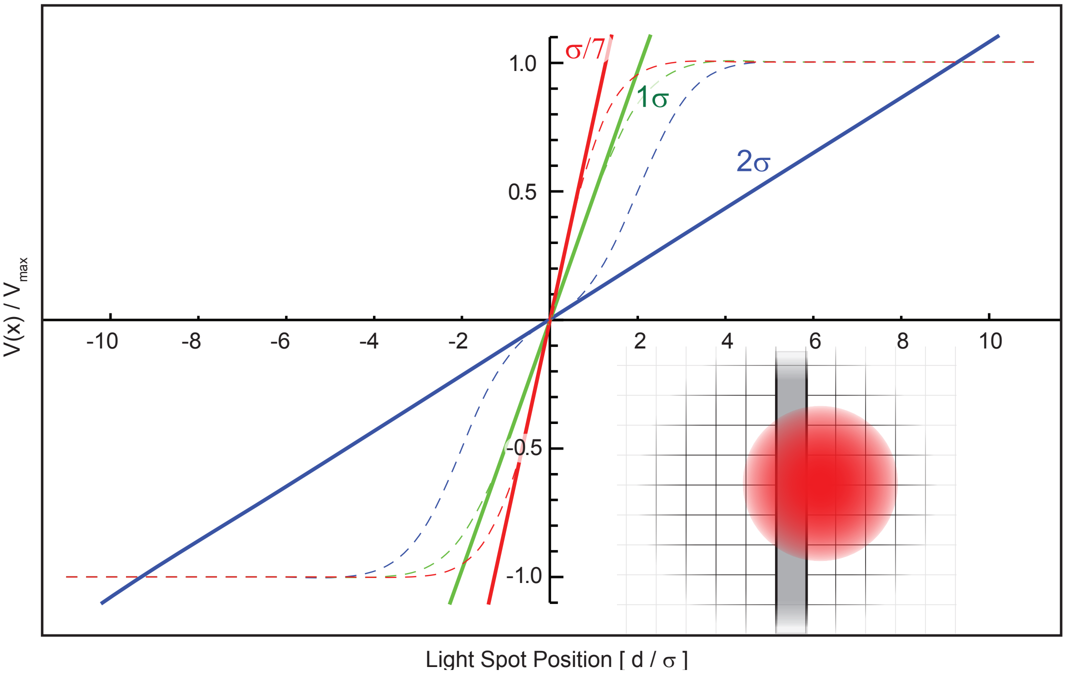

Figure SF4. Signal versus displacement for a split detector of infinite size and insensitive gap between segments of half width $\delta = 1/7\sigma, 1\sigma, 2\sigma$ in red, green and blue. Exact solution in segmented line, and linear approximation in solid line. The effect of the insensitive gap size becomes considerable when $\delta > \sigma/7$.

SS2 Diffraction model

We model the optical system using the Huygens-Fresnel principle, where the light re-emitted by the cantilever, both reflected or transmitted, can be understood as the summation of an infinite number of infinitesimal point sources located on the cantilever surface.

A cantilever acts as a rectangular slit source in the plane (ξ,η) and under Fresnel's approximation the optical wave in the observing plane (x,y) is given by[1]

$$U(x,y) = \frac{e^{ikz}}{i\lambda z}\iint_{-\infty}^{\infty} U(\xi,\eta,z)\, e^{i\frac{\pi}{\lambda z}\left[(x-\xi)^2+(y-\eta)^2\right]} d\xi d\eta. \tag{SE15}$$

where $U(\xi,\eta,z)$ is the function defining the amplitude and phase at the re-emitting source, defined by the illuminating profile and the geometry of the device.

To model the influence of a finite cantilever we first separate variables using $U(x,y,z) = -i\exp(ikz)\mathscr{I}(x)\mathscr{I}(y)$ and concentrate attention on the longitudinal axes x, ξ.

SS2.1 Cantilever edge

We now introduce an edge at position $\xi = s$, and re-define $U(\xi) = I'\,H(\xi - s)\,\exp(-\frac{1}{2}(\frac{\xi}{\sigma})^2)$, where $H(x)$ is the Heaviside step function, $I' = I_o/\sigma\sqrt{2\pi}$ to obtain:

$$\mathscr{I}(x) \quad = \quad \frac{I'}{\sqrt{\lambda z}}\int_{-\infty}^{s} e^{\frac{\pi i}{\lambda z}(x-\xi)^2}\, e^{-\frac{1}{2}\left(\frac{\xi}{\sigma}\right)^2} d\xi\,, \tag{SE16}$$

where ξ is the coordinate along the cantilever, x along the detector, λ is the illumination wavelength, s the position of the cantilever edge and σ the standard deviation of the Gaussian beam.

$$\frac{1}{4}\exp\left(-\frac{(\sigma+x)^2}{\frac{\lambda z^2}{16\pi^2\sigma^2}+\sigma^2}\right)\left\|\frac{\left(\operatorname{erf}\left(\frac{\lambda z+4i\pi x\sigma}{\sqrt{2}\sqrt{\lambda z(\lambda z-4i\pi\sigma^2)}}\right)+1\right)^2}{\lambda z-4i\pi\sigma^2}\right\| \tag{SE17}$$

where erf is the Error function.

SS2.2 Curved cantilever

We assume homogenous illumination over the whole cantilever.

SS2.2.1 Reflection

We have previously shown that we can describe the boundaries on amplitude and phase of the electromagnetic wave imposed by the surface of a rectangular cantilever of dimensions (w,l) curved along the ξ axis by defining[2]

$$U(\xi,\eta) = S_\xi S_\eta \exp\left[\frac{4\pi i}{\lambda}\left(a\xi + b\xi^2\right)\right]; \tag{SE18}$$

where $S_\xi = \mathrm{rect}(\xi/l)$, $S_\eta = \mathrm{rect}(\eta/w)$ define the dimensions of the cantilever, a and b are the coefficients of the quadratic shape that describes the cantilever tilt and curvature and the exponential in the right-hand-side models the phase from the difference in optical path caused by the bending of the surface. By rearranging equation (SE16) for a perfect square binomial we can write

$$\mathscr{I}(x) = A\int_{-\infty}^{\infty} S_\xi \exp\left[i\frac{\pi}{\lambda z}\left(\frac{x-2az}{1+4bz}-\xi\right)^2\right] d\xi; \tag{SE19}$$

where $A = (\lambda z)^{-\frac{1}{2}} \exp\left[i\frac{4\pi}{\lambda\,(1+4bz)}\left(bx^2+ax-a^2z\right)\right]$. We define

$$x' = \frac{x-2az}{1+4bz} \tag{SE20}$$

and observe that the term $2az$ causes a shift and $1+4bz$ a magnification of the diffraction pattern.[2] Applying the condition $z \gg \xi^2/\lambda$ the Fraunhofer approximation for the far field $\exp(2\pi i\xi^2/\lambda z) \approx 1$ implies

$$\mathscr{I}(x) \approx Ae^{i\frac{\pi}{\lambda z}x'^2} \int_{-\infty}^{\infty} S_\xi \exp\left[i\frac{\pi x'}{\lambda z}\xi\right] d\xi, \tag{SE21}$$

and therefore the observed intensity profile is given by

$$|\mathscr{I}(x)|^2 \approx \frac{1}{\lambda z}\left|\int_{-\infty}^{\infty} S_\xi \exp\left[2\pi i\left(\frac{x'}{2\lambda z}\right)\xi\right] d\xi\right|^2. \tag{SE22}$$

SS2.2.2 Transmission

To model the transmission mode, we took a step back and considered the Fresnel approximation not in a plane but from a curved source. According to the Huygens-Fresnel principle the field intensity is given by[1]

$$U(x,y,z) = \iiint \frac{z}{i\lambda r^2} U(\xi,\eta,\zeta) \exp\left(\frac{2\pi i}{\lambda}r\right) d\xi d\eta d\zeta \tag{SE23}$$

where $r = \sqrt{(x-\xi)^2+(y-\eta)^2+(z-\zeta)^2}$ is the distance from the virtual source point at (ξ,η,ζ) and the observation at (x,y,z). We consider a source curved on ξ, $U(\xi,\eta,\zeta) = S_\xi S_\eta \delta(\zeta - a\xi - b\xi^2)$ and follow similar procedure as before but with z replaced by $z+a\xi+b\xi^2$. We approximate r up to second order in the numerator exponent and re-arrange to complete squares

$$r \approx A_{\mathrm{Tr}} + \frac{m}{2z}\left(\xi - \frac{x-n}{m}\right)^2 \tag{SE24}$$

where the zero order term A_{Tr} will later cancel with its complex conjugate when calculating the intensity and

$$n = az - a\frac{x^2}{2z} \tag{SE25}$$

$$m = 1+2bz+2a\frac{x}{z} - b\frac{x^2}{z} \tag{SE26}$$

To the extent that $x \ll z$ we can neglect the terms containing x in the right hand side and recover a similar result as before, this time only for small changes in cantilever tilt and curvature, implying a pattern shift of az, pattern magnification of $1+2bx$ respectively. These magnitudes differ from the previous result by a factor of two because, for a given cantilever displacement, light travels the path only once in transmission mode but twice in reflection mode.

References

1. Goodman, J. W. *Introduction to Fourier optics* (Roberts & Company Publishers, 2005), 3 edn. URL https://books.google.co.uk/books?id=ow5xs_Rtt9AC.
2. Hermans, R. I., Bailey, J. M. & Aeppli, G. Direct and alignment-insensitive measurement of cantilever curvature. *Appl. Phys. Lett.* **103**, 34103–34105 (2013). URL http://dx.doi.org/10.1063/1.4813265.
